# Effect of Sodium Flouride Mouthwash and Ozone-Infused Oil Pulling Solution With Coconut Oil on Mechanical Properties and Surface Characterization of Copper-Nickel-Titanium Orthodontic Archwires

**DOI:** 10.7759/cureus.40207

**Published:** 2023-06-10

**Authors:** Buddha Sukumar, RSVM Raghu Ram, Ghanta Sunil, Inuganti Ranganayakulu, Kuchimanchi Anand Viswanadh

**Affiliations:** 1 Orthodontics & Dentofacial Orthopaedics, GSL Dental College & Hospital, Rajahmundry, IND

**Keywords:** surface characteristics, mechanical properties, o3, naf, cu-ni-ti

## Abstract

Aims and objectives: To determine the impact on the mechanical properties and surface characteristics of prefabricated 0.016" copper-nickel-titanium (Cu-Ni-Ti) type 35^o^C (Ormco Company, USA) archwires when subjected to 0.05% sodium fluoride (NaF) mouthwash (ACT Anti-Cavity Fluoride Mouthwash, Sanofi Company, USA) and ozone-infused oil-pulling solution with coconut oil (O_3_) (O_3_ Essentials, Health Ranger Store, USA).

Materials and methods: Sixty samples of preformed maxillary 0.016” Cu-Ni-Ti archwires were cut at the straight posterior ends for a length of 25 mm and then equally distributed into three groups (n=20). Each group of wires was immersed in distilled water (dH_2_O), NaF, and O_3_ solutions for 90 minutes at 37^o^C. All samples were taken out of their solutions and washed with distilled water prior to testing. On a universal testing device, a three-point bending test was performed on 15 samples. Yield strength (YS), flexural modulus of elasticity (E), and springback ratio (YS/E) were calculated. The remaining five samples from respective solutions were observed under a scanning electron microscope (SEM) for surface topography evaluation.

Results: The mean differences in loading YS, E, and YS/E between NaF and O_3_ are 41.14 MPa, 4.58 GPa, and -0.0006 whereas unloading values are 23.45 MPa, 4.38 GPa, and -0.0004, respectively with a statistical significance of <0.001. Surface topography alteration was appreciated in the NaF mouthwash group compared to the O_3_ solution.

Conclusions: The mechanical properties of 0.016" Cu-Ni-Ti archwires during loading and unloading were changed after exposure to NaF mouthwash and O_3_ solution. The mechanical properties of Cu-Ni-Ti archwires were more negatively affected by NaF mouthwash than by O_3_ solution. Sodium fluoride mouthwash offers more corrosive changes when compared with the O_3_ solution.

## Introduction

Fixed orthodontic appliances considerably increase plaque accumulation, alter the gingival and plaque index, and impede oral hygiene [1]. Most orthodontists recommend their patients to use fluoride-containing regimens additionally along with daily brushing and flossing procedures during orthodontic treatment to prevent tooth decay [2]. While there are numerous commercially available antimicrobial mouthwashes on the market, sodium fluoride (NaF) is the most widely used rinsing agent and has some negative effects on orthodontic materials [3]. During ancient times some ayurvedic procedures like oil pulling were likely used as an adjuvant for oral hygiene practices [4]. Oil-pulling therapy involves some oils like coconut oil, olive oil, sesame oil, etc., which were advised for mouth rinsing in the early mornings on an empty stomach for 20 minutes [5]. Incorporating ozone into mouth rinsing has recently been shown to be more helpful than traditional therapy methods, which adopt a minimally invasive and conservative approach to dental treatment and are inexpensive [6]. There was strong proof that human oral epithelial cells, periodontal cells, and gingival fibroblast were biocompatible with ozonated oil [7]. Many studies explained the anti-microbial action of ozone-infused oral rinses but, none of the studies explained their action on fixed orthodontic appliances, especially the archwires which are placed over a long span. In this study, NaF and ozone-infused oil-pulling solution with coconut oil (O_3_) mouth rinsing agents were preferred over the other commercially available rinses due to their good resistance to bacterial count, unique properties, and beneficial effects. All orthodontic archwires were utilized to project forces, but copper-nickel-titanium (Cu-Ni-Ti) archwires produce forces that are more accurate and consistent over a longer duration. Due to its availability at several phase transformation temperatures (15^o^C, 27^o^C, 35^o^C, and 40^o^C), the clinician can choose the appropriate archwire for each individual situation [8]. Due to the long interval stay of these wires in patient’s mouths, and exposure to mouth rinses, there could be changes in the wire characteristics. There was little information available on the potential effects of mouthwashes over Cu-Ni-Ti archwires. As a result, the goal of this in-vitro investigation is to assess how 0.05% NaF mouthwash and O_3_ solution affect the mechanical properties and surface characterization of Cu-Ni-Ti archwires.

## Materials and methods

Methodology

Considering the preliminary data acquired, power analysis at 80%, and fixing the confidence level at 95%, a sample size of 60 was fixed for this study. A total of three groups (n=20 each) meet the constraints of α = 0.05. Of the 20 samples in each group, 15 samples were for determining the mechanical properties, and the rest five were for observing the surface characteristics of the archwires when subjected to various mouth rinses. Review board clearance from the GSL Educational Institution was obtained for the use of the study (GSLEI/IRB/2019/003). Sixty samples of 0.016” preformed maxillary Cu-Ni-Ti type 35^o^C (Ormco Company, USA) archwires were cut from the posterior part of the straight ends for a length of 25 mm. These wires were randomly distributed into three groups. Each group was incubated at 37^o^C in a separate sterile petri dish with 10 mL of 0.05% NaF mouthwash (ACT Anti-Cavity Fluoride Mouthwash, Sanofi Company, USA), O_3_ (O_3_ Essentials, Health Ranger Store, USA) and distilled water (dH_2_O) (control solution) for 90 minutes in an incubator. According to Mary P Walker et al., (2005), this period of time would be comparable to three months of using topical fluoride or fluoride mouthwashes for one minute each day [9]. Before being put through mechanical testing, samples from the various experimental groups were cleansed with dH_2_O and transferred to new containers with labels for their respective groups. A three-point bend test was performed on all the samples on a universal testing machine (UTES-40-HGFL) with a 5 kN load cell. On the machine, the two poles were spaced 12 mm apart in the lower jaw, which simulated the inter-bracket distance between the teeth. A steel rod with a bevel end chisel was positioned halfway between the two poles and a compressive force at a crosshead speed of 0.5 mm/min was applied. Each sample was loaded to a deflection of 3.1 mm and then unloaded to zero deflection at a crosshead speed of 2.5 minutes [9]. Using computer software, each sample's load in newtons (N) and deflection in millimeters were recorded during both loading and unloading. The data was used to produce load-deflection curves, and the engineering beam theory was applied to determine the yield strength (YS) and the flexural modulus of elasticity (E). The springback ratio (YS/E) was calculated based on the data obtained [10]. The loading curves obtained from the three-point bending test simulate the activation of the wire, whereas the unloading curves depict deactivation forces which provide an indication of its potential clinical behaviour.

Evaluation of surface characterization

The surface properties of the archwires were evaluated using a scanning electron microscope (SEM) (Hitachi, S-3700N) at an x1000 magnification. Each sample was put on a holder and examined with a field-emission SEM device. The archwire surface was bombarded with electrons, and each pixel on the SEM image was examined for the electron reflection intensity. Based on a visual assessment of the surface irregularities, the surface topographical characteristics were established [9].

The obtained information was computed using IBM Statistical Package for Social Sciences (SPSS) version 20 (Armonk, NY, USA) software. The statistical analysis carried out included the means and standard deviations of all the variables. Kolmogorov-Smirnov test was used to investigate the normality of the data obtained. One-way analysis of variance (ANOVA) was done with a 5% level of significance (α =.05) for both the loading and unloading of the archwire. YS, E, and YS/E for each sample were recorded and compared to see if there were any notable variations between the groups. Tukey’s post hoc tests for multiple pairwise comparisons were done to analyze the data.

## Results

Normality in the distribution of data was observed in regard to the parameters (Table 1).

**Table 1:** Distribution of data with regard to all the study parameters

Parameter		Kolmogorov-Smirnov statistic	Df	P value
Loading	Yield strength (MPa)	0.1	45	0.2
	Modulus of elasticity (GPa)	0.106	45	0.2
	Springback ratio	0.099	45	0.2
Unloading	Yield strength (Mpa)	0.114	45	0.17
	Modulus of elasticity (GPa)	0.106	45	0.2
	Springback ratio	0.106	45	0.2

On mechanical testing, it was noted that all three groups, including the control, displayed statistical significance for both loading and unloading features (Table 2).

**Table 2:** Comparison of loading and unloading mechanical properties between control and experimental group One-way analysis of variance (ANOVA) was performed to compare the mechanical parameters of loading and unloading between the experimental and control groups

Parameter		Distilled water	Ozone-infused oil-pulling solution with coconut oil	Sodium fluoride mouthwash
Yield strength (MPa)	Loading	1188.85 ± 22.71	1146.77 ± 23.16	1105.63 ± 26.11
	Unloading	683.59 ± 13.06	653.65 ± 13.2	630.2 ± 14.88
Modulus of elasticity (GPa)	Loading	70.08 ± 2.67	65.2 ± 2.63	60.02 ± 2.84
	Unloading	66.9 ± 2.55	62.27 ± 2.51	57.8 ± 2.71
Springback ratio	Loading	0.0016 ± 0.0003	0.0017 ± 0.0003	0.0018 ± 0.00043
	Unloading	0.0102 ± 0.0002	0.0105 ± 0.0002	0.0109 ± 0.00026

The data were analyzed using Tukey's post hoc testing for multiple pairwise comparisons. The results from the control group showed a significant difference between the O_3_ solution and NaF, indicating that both mouthwashes reduced the mechanical properties of Cu-Ni-Ti wires during loading and unloading (Table 3).

**Table 3:** Multiple pairwise comparisons of mechanical properties on loading and unloading between the study groups Tukey's post hoc analysis was used to compare mechanical parameters during loading and unloading between the research groups for pairwise comparisons; * denotes statistical significance

Parameter	Reference group	Comparison group	Loading		Unloading	
			Mean difference	P value	Mean difference	P value
Yield strength (MPa)	Distilled water	Ozone-infused oil-pulling solution with coconut oil	42.08	<0.001*	29.93	<0.001*
		Sodium fluoride mouthwash	83.22	<0.001*	53.38	<0.001*
	Ozone-infused oil-pulling solution with coconut oil	Sodium fluoride mouthwash	41.14	<0.001*	23.45	<0.001*
Modulus of elasticity (GPa)	Distilled water	Ozone-infused oil-pulling solution with coconut oil	4.87	<0.001*	4.65	<0.001*
		Sodium fluoride mouthwash	9.45	<0.001*	9.03	<0.001*
	Ozone-infused oil-pulling solution with coconut oil	Sodium fluoride mouthwash	4.58	<0.001*	4.38	<0.001*
Springback ratio	Distilled water	Ozone-infused oil-pulling solution with coconut oil	-0.0006	<0.001*	-0.0002	0.004*
		Sodium fluoride mouthwash	-0.0013	<0.001*	-0.0006	<0.001*
	Ozone-infused oil-pulling solution with coconut oil	Sodium fluoride mouthwash	-0.0006	<0.001*	-0.0004	<0.001*

## Discussion

Demineralization is a natural side effect of fixed orthodontic therapy, particularly when it is combined with bad oral hygiene. White spot lesions and consequent enamel demineralization are caused by the acidic byproducts of the bacterial consortia in plaque [11]. Orthodontists commonly prescribe daily topical fluoride to prevent plaque accumulation, and demineralization of tooth structure [2]. However, these prophylactic agents get in constant touch with the components of fixed orthodontic appliances like brackets, archwires, etc., which in turn results in the disruption of the titanium oxide layer on nickel-titanium alloys. This leads to changes in the mechanical properties and surface of the archwire, as the alloy loses its passivating effect and hydrogen embrittlement, thereby hindering the treatment outcome of the orthodontic archwires [12]. The current study findings revealed that dH_2_O has a statistically significant (<0.001) difference in terms of higher YS, E, and YS/E when compared to NaF mouth rinse. However, M Kaushik et al., (2011) observed a significant change only in the unloading mechanical properties between NaF mouth rinse and dH_2_O [5]. They stated that the difference in YS may be due to NaF and its additives which may cause the deterioration of Cu-Ni-Ti archwires. The change in the E may be due to titanium hydride formation at interstitial regions of a crystal lattice. Additionally, they noted that titanium hydrides were thought to create a body-centered tetragonal structure, which was the primary factor altering the alloy's mechanical properties. The present study agrees with the reasoning given by M Kaushik et al., (2011) regarding the change in mechanical properties of Cu-Ni-Ti archwires when exposed to NaF compared with dH_2_O [5]. A similar explanation was given by Mary P Walker et al. (2005) for both loading and unloading forces but there was no statistically significant difference in the mechanical properties mentioned [9]. They claimed that when Ni-Ti and Cu-Ni-Ti archwires are subjected to fluoride rinses, hydrogen absorption and embrittlement of titanium-based alloys are detected. Titanium typically has a surface oxide layer that blocks hydrogen from penetrating and prevents the creation of ionizable fluoride compounds, but when fluoride elements like sodium fluoride and hydrogen fluoride are exposed, this layer is activated, which leads to fast corrosion. The addition of Cu in Cu-Ni-Ti wires serves as a relative inhibitor of reducing acids like hydrogen fluoride. As a result, less titanium hydride will form in the lattice structure, which will help to keep the wire's mechanical properties from changing. Distilled water didn’t have any fluoride agents in it; so, it offers less alteration in mechanical properties of Cu-Ni-Ti archwires when compared with fluoride rinses. In contrast to the findings of the present work, Arthi Ramalingam et al., (2008) showed that after immersing Cu-Ni-Ti in dH_2_O and NaF mouthwash, there was no significant difference in YS and E considering both loading and unloading forces [13]. In the present study, when the mean values are compared it shows that both in loading and unloading, dH_2_O offers more YS, E, and YS/E with a statistical significance of <0.001. There haven't been any contradictory studies on the influence of O_3_ and dH_2_O on the mechanical characteristics of Cu-Ni-Ti archwires under loading and unloading stresses up to this point. When compared with dH_2_O, there is a higher degree of change observed in the mechanical properties during loading and unloading forces of Cu-Ni-Ti archwires after immersion in O_3_ was observed. Generally, ozone is an easily soluble oxidizing molecule that can be dissociated into unstable nascent oxygen and oxygen molecule; so, ozone is delivered by means of ozonated water or oil forms. L Zhu et al., (2004) stated that when titanium reacts with oxygen, it forms a titanium dioxide (TiO_2_) layer at more than 400^o^C [14]. Moreover, the TiO_2_ layer acts as a protective layer which prevents further corrosion. However, In the current study, Cu-Ni-Ti archwires are subjected to ozone-infused coconut oil pulling solution at 37^o^C only; so, ozone might not alter Cu-Ni-Ti archwire mechanical properties. The change in the mechanical properties may be because of other ingredients which are present in ozone-infused coconut oil pulling solution. Moreover, the effect of coconut oil on fixed orthodontic materials was nowhere elucidated in the literature to date. In the present study, when the mean values are compared, it shows that both in loading and unloading, O_3_ offers more YS, E, and YS/E with a statistical significance of <0.001. There are no confounding articles present regarding the comparison of mechanical properties of Cu-Ni-Ti archwire after immersion in O_3_ and NaF mouthwash.

**Figure 1:**
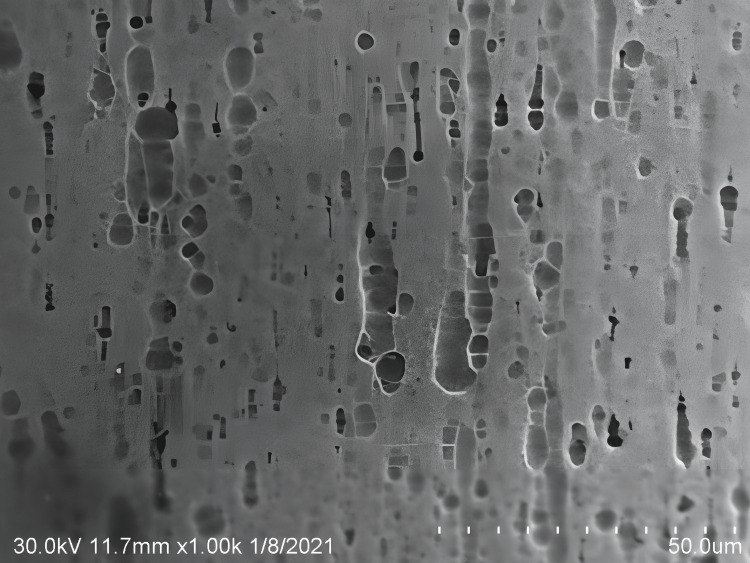
Surface topography of copper-nickel-titanium wires exposed to distilled water

An SEM was used to determine the qualitative assessment of constituent elements in the alloys [15]. When viewed at x1000 magnification in SEM during the current study, the archwire surface exposed to dH_2_O appeared to have a lot of dark spots on the Cu-Ni-Ti wire surface (Figure 1).

The current study's findings agreed with those of M Kaushik et al., (2011) and Mary P Walker et al., (2005) [5, 9]. They claimed that the coating of byproducts applied to the Cu-Ni-Ti and Ni-Ti archwires during the production process may have been the cause of the alteration in the surfaces of those materials.

**Figure 2:**
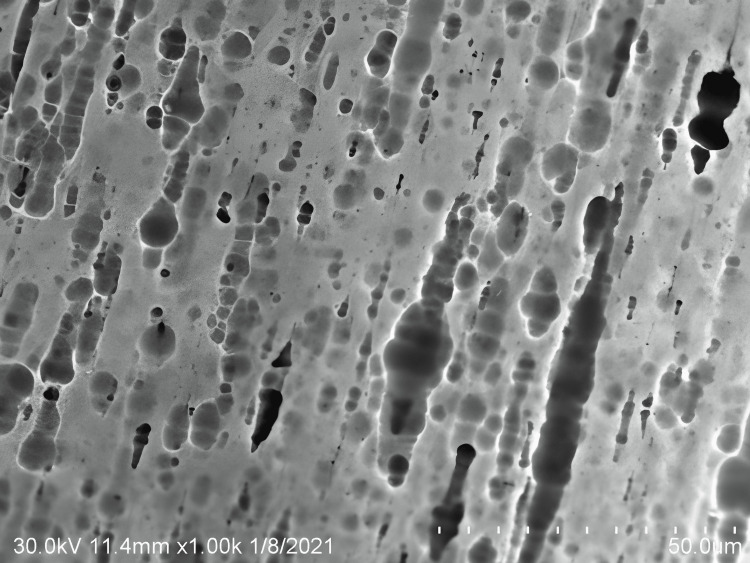
Surface topography of copper-nickel-titanium wires subjected to sodium fluoride mouthwash

Cu-Ni-Ti archwires when subjected to NaF mouth rise observed in SEM at x1000 magnification exhibit an enlarged bright white patch with globular, pitted, and elongated flaws and a much larger exposure of white inclusions (Figure 2).

The findings of the present study were in accordance with M Kaushik et al., (2011) [5]. The investigation by Mary P Walker et al., (2005) observed that pitting emerges along the wire's wrought surface and noted that the pH and fluoride concentration of the preventive agent affects the fluoride-related alloy [9]. According to the findings of the current study, the components of NaF mouthwashes (sodium or fluoride) were to blame for the disintegration of the titanium oxide layer on Cu-Ni-Ti arch wires, which resulted in corrosive alterations.

**Figure 3:**
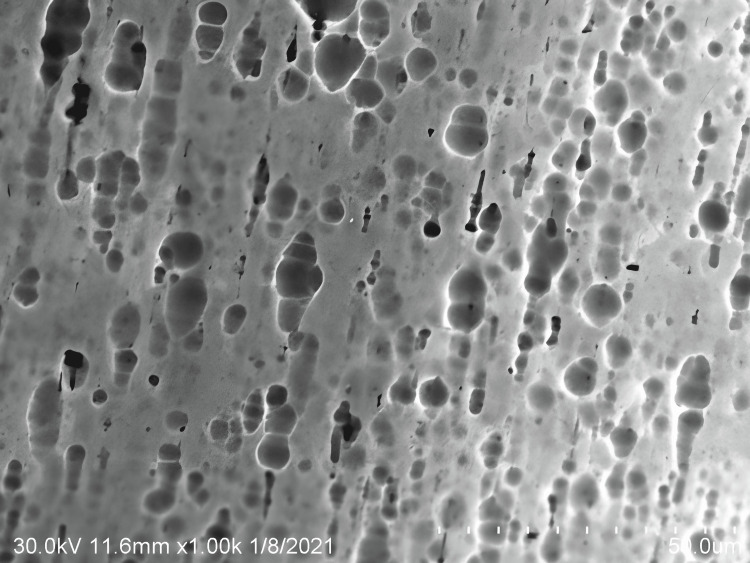
Surface topography of copper-nickel-titanium wires subjected to ozone-infused oil-pulling solution with coconut oil

A mottled, pitted appearance in addition to dark smudge patches was seen at x1000 magnification under an SEM for the Cu-Ni-Ti archwires after being submerged in an O_3_ solution (Figure 3).

In the present study after immersion of Cu-Ni-Ti archwire in NaF, the surface of the archwire showed bright white spots, pitting with appreciated defects that were not clearly observed in dH_2_O. In the current study, exposure to NaF resulted in more corrosive changes than in the control group. Comparing the experimental groups, after immersion of Cu-Ni-Ti archwire in NaF mouthwash, the archwire exhibited pitting with clearly demarcated bright white spots with dark smudges. However, these findings are less observed in O_3_ solutions.

Limitations

As this is an in vitro investigation, the results cannot be directly compared to clinical performance. Variations in the experimental conditions might differ in the shear bond strength (SBS) values. Mechanical properties and surface characteristics of Cu-Ni-Ti archwires might differ from in vivo to in vitro.

Further scope

As the oral conditions influence the mechanical properties and surface characteristics, further clinical studies are necessary for validation, especially for O_3_ solutions.

## Conclusions

On exposure to sodium fluoride mouthwash and ozone-infused oil-pulling solution with coconut oil, the mechanical properties during loading and unloading of 0.016” copper-nickel-titanium archwires were altered. Sodium fluoride mouthwash showed more deleterious changes in the mechanical properties of copper-nickel-titanium archwires while ozone-infused oil-pulling solution with coconut oil showed lesser changes. Sodium fluoride mouthwash offers more corrosive changes when compared with the ozone-infused oil-pulling solution with coconut oil.
